# A discrete transition zone between cuticle and cortex layers of a human hair fibre: changes observed in the presence of breast cancer

**DOI:** 10.3332/ecancer.2017.772

**Published:** 2017-10-13

**Authors:** Donald Lyman, Paula Gerstmann

**Affiliations:** 1 Department of Bioengineering, University of Utah, Salt Lake City, Utah 84112, USA; 2 Zeitig Research, Olympia, Washington 98506, USA

**Keywords:** human hair, ATR FT IR spectroscopy, breast cancer, discrete transition zone, lipid esters

## Abstract

Attenuated total reflection Fourier transform infrared (ATR–FT-IR) spectroscopy of hair fibres shows a discrete transition zone (DTZ) between the hard protective cuticle layer and the softer elongated cortical cells of the cortex. The DTZ is composed of flattened orthocortical cells located on the outer perimeter of the cortex and appears to range in thickness between 2 and 3.6 μm. The inner surface of the DTZ, adjacent to the elongated cortical cells that make up the core of the hair fibre, is irregular. ATR–FT-IR analyses of these flattened orthocortical cells indicate major changes in the molecular structure of keratins found in this transition zone. Other studies have identified cells that produce keratins that are distinct from alpha keratins found in the elongated heterocortical cells in the hair fibre core. These distinct keratins appear to be produced in the lower region of the hair follicle at the interface between the cuticle and cortex.

The DTZ is also the region where ATR–FT-IR spectroscopy studies identified changes in C−H bending of lipid esters indicative of breast cancer. Lipid ester absorption bands at 1738 and 1732 cm^−1^, present in non-cancer hair, are absent in the cancer hair and a new ester band absorbing at 1736 cm^−1^ is observed. When the breast cancer is clinically removed, the 1736 cm^−1^ ester band absorption and the increase in the 1446–1456 C−H-bending absorption ratio are no longer observed. This suggests that biomarkers produced by the breast cancer interact with stem or other cells near the lower region of the follicle, controlling the amount and type of lipid esters in the DTZ.

## Introduction

The hair follicle can be considered a miniorgan with its main function being the production of a hair fibre [1]. The hair fibre is a layered structure formed in the bulb area of the follicle where a complex, integrated system of molecular synthesis, polymerisation, cell formation and changes in the physical organisation of these molecules occur. The external part of hair fibre is essentially stable dead tissue. Consequently, the complex process of its formation has been studied primarily in terms of its commercial use as textile fibre, such as wool, or for the cosmetic treatments of human hair.

James first reported the subtle changes detected in the synchrotron X-ray diffraction patterns of human hair resulting from breast cancer [2]. Hair from cancer patients showed one or more diffraction rings of comparatively low intensity superimposed at specific locations on the normal hair alpha keratin pattern, whereas hair from non-cancer patients did not. It was reported that with careful attention to precise techniques, high-energy synchrotron X-ray diffraction can reproducibly detect clear and consistent changes in the hair of breast cancer patients [3–7]. These studies have advanced to clinical trials, with initial results showing that an altered X-ray diffraction pattern indicates the presence of breast cancer with an overall accuracy of greater than 77% [8].

Changes in the X-ray diffraction patterns of hair from breast cancer patients suggested that compositional changes have occurred in the hair structure. This prompted our studies to determine the underlying molecular and conformational changes in the hair fibre using infrared spectroscopy. Fourier transform infrared (FT-IR) spectroscopy, with its high-resolution and high-energy throughput over the entire spectral region and a good signal to noise ratio, enables the study of both molecular (primary) and conformational (secondary) structures of biological molecules. Attenuated total reflection (ATR) techniques also enable the analysis of hair in a relatively non-destructive manner. An initial study of the ATR–FT-IR spectra of hair samples from non-cancer and cancer patients [9], using hair samples supplied to us by James, indicated that spectra from breast cancer patients showed increased C−H bending absorptions in the 1500 to 1400 cm^−1^ region compared to non-cancer individuals. This supported her postulate that the superimposed rings on the normal alpha keratin X-ray diffraction patterns were from lipid materials [2]. Later ATR–FT-IR studies suggested that this increased lipid material is primarily located in cuticle-cortex interface area [10, 11]. To determine more precisely the location and the nature of this increase in lipid material, a more complete compositional analysis of this region of the hair fibre structure was undertaken using variable angle ATR FT IR spectroscopy.

In this paper, we present our variable angle ATR-FT-IR analysis of the interface between the cuticle and cortex regions of hair fibres and propose that it is distinct from those two regions and is altered by the presence of breast cancer.

## Methods

Samples of hair from 12 female Caucasian subjects previously studied [11] were re-examined using variable angle methods to study the interface region between the cuticle and cortex layers of the hair fibre. Three hairs from each individual were mounted on their own sample card so that the section of hair fibre 5 mm from the scalp end was positioned parallel to the infrared beam. The sample cards were then stored over anhydrous CaSO_4_ in a desiccator jar for 48 h before the hair spectra were obtained.

ATR–FT-IR absorption spectra were obtained from 4000 to 700 cm^−1^ using 128 scans at a resolution of 4 cm^−1^ and Norton–Beer medium apodisation using a Thermo Nicolet Nexus 670 spectrometer with a liquid N2-cooled mercury cadmium telluride (MCT) detector. The ATR cells used in this study were a SplitPea accessory with a zinc selenide 45° internal reflection element (IRE) and a modified SeaGull^TM^ with a zinc selenide (ZnSe) IRE (both from Harrick Scientific, Pleasantville, NY).

The Grams 386 program (Galactic Industries, Salem, NH) was used for spectral manipulations. Peak heights and integrated areas were determined from spectra that were baseline corrected and water subtracted.

**Calculations:** The penetration depth (*d*_p_) of the infrared beam into the sample, defined as the distance for the electric field amplitude to fall to e^−1^ of its value at the surface, was calculated from Harrick’s equation [12]: *d*_p_ = λ / 2 π ηc[sin^2^θ – (ηs/ηc)^2^]^½^, where θ is the angle of incident light, λ is the frequency of the infrared beam, ηc is the refractive index of the internal reflection element (IRE), and ηs is the refractive index of the sample. The refractive index of hair (ηhair) is reported to be 1.555 [13]. The refractive index of ZnSe is 2.42.

The effective penetration depth (or sampling depth, de) of the evanescent wave has also been shown to increase as the ratio of the refractive index of the sample to the refractive index of the IRE approaches 1.0 [14, 15]. For KRS5 (refractive index of 2.4) with a sample/IRE ratio of 0.63, *d*_e_ approximated 3*d*_p_. One would expect a similar effect for ZnSe IRE and hair (refractive index of 1.555) since the sample/IRE ratio (0.648) is similar. Since *d*_e_ also increases as the IR frequency increases, optimal incident light angles to be used for each frequency range must be determined so that the spectrum represents material found at a similar concentric layer depth in the hair fibre. These *d*_e_ values are tabulated in Table 1.

## Results and discussion

Formation of the hair fibre occurs in the hair follicle, an epidermal tube dilated at the base to form a bulb [1, 16–20]. The dermal papilla, located at the base of the bulb where the blood supply comes into the follicle, controls much of the biological signalling from the blood to the specialised fibroblast cells located internally in this area of the bulb. Key to the formation of the hair fibre are matrix stem cells located around the dermal papilla that initiate the production of the cellular and molecular materials. To form a human hair fibre of the structural complexity seen in X-ray diffraction, transmission electron microscopy, and ATR–FT-IR spectroscopy, one expects that each area of the hair fibre structure requires its own specialised stem cells [20–23]. The bulb and the lower part of the follicle tube is where differentiation and biological synthesis of cells and molecules occur, followed by the radial organisation of these constituents into the preliminary hair fibre structure and the supporting inner root sheath. Since hair grows about 0.4 mm/day and the hair fibre is completely formed when it reaches approximately 1 mm from the matrix cells in the follicle bulb, it takes about 2.5 days for hair to be fully formed.

The continuous production of these organised cells provides the materials to form the hair fibre and creates the axial forces needed to force the hair fibre substrate along the narrowing tubular structure of the hair follicle. The increased radial forces from the narrowing follicle structure also cause the cuticle cells to be flattened against the internal root sheath and interlocking with it and cause the central core of cortical cells to become elongated. During the cellular elongation, the conformation of the keratin macromolecules in the cortical cells become physically altered from random chain structures into elongated chain structures. These dimerise to form coiled-coil structures and further aggregate into more complex intermediate filament and macrofibril structures. This results in the keratinisation process and the formation of the final hair fibre with the macrofibrils aligned along the axis. This macrofibril structure gives rise to the alpha keratin X-ray diffraction pattern of the hair fibre cortex [24–26]. At this latter stage, the hair fibre is essentially dead tissue and thus provides a permanent record of any prior synthesis and processing events.

As shown by James [2–6], in the presence of breast cancer the X-ray diffraction pattern of the hair fibre changes, showing a faint ring pattern superimposed on the normal alpha keratin pattern. Attenuated total reflection (ATR) infrared spectroscopy techniques also enable the analysis of a hair fibre in a relatively intact and non-destructive manner. An initial study of the ATR FT-IR spectra of hair samples from non-cancer and cancer patients [9] using hair samples supplied to us by James, indicated that spectra from breast cancer patients showed increased C−H bending absorptions in the 1560 to 1440 cm^−1^ region compared to non-cancer individuals. This supported James’s postulate that the superimposed rings on the X-ray diffraction patterns were from lipid materials [2]. Later, ATR FT-IR studies suggested that this increased lipid material indicating the presence of breast cancer is located in the interface region between the cuticle and the cortex.

**Figure 1.**
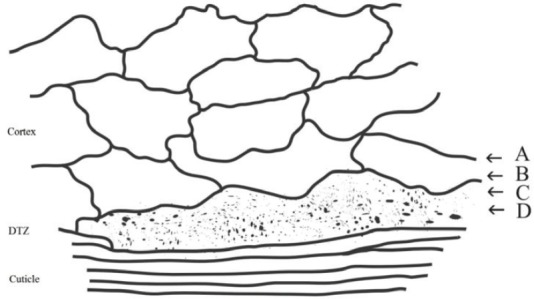
Line drawing of TEM pattern of cuticle–cortex interface [17] showing the area that would provide a discrete transition zone (DTZ) between the cuticle and cortex. The arrows indicate approximate location of spectral slices A, B, C and D described in the discussion below.

The ultrathin electron microscopic section of human hair by Kassenbeck (Figure 16 in [17]) shows a region of flattened orthocortical cells adjacent to the cuticle that are different from the heterotype cortical cells inside the main body of the cortex. They are more flattened, show a decrease in sulphur content and stain deeper than the bulk of the cortex, that is, the heterotype cortical cells [17, 27]. A line drawing of this electron microscope thin section photo [17] is shown in our Figure 1. This area of the flattened orthocortical cells would be expected to provide a discrete transition zone (DTZ) between the hard protective cuticle layer and the softer elongated cells of the cortex. Because of the irregularity of its inner surface, this DTZ appears to range from 2 to 3.6 μm in thickness.

To investigate the structure of the DTZ in more detail, further analyses were conducted using breast cancer positive hair samples. The Harrick SeaGull^TM^ variable angle ATR FT-IR cell with a spherical ZnSe IRE was used to obtain the variable angle spectra of these hair fibres. Spectral slices of the hair fibre were then obtained by subtractions of the various θ spectra using the Grams Subtract AB Auto-Factor program. Spectral subtractions were done using the 1760–1700 cm^−1^ region for ester absorptions, the 1700–1610 cm^−1^ region for Amide I absorptions, and the 1500–1400 cm^−1^ region for the C–H bending absorptions. Approximate locations of these spectral slices in the hair fibre structure are shown in Figure 1. The data for a hair fibre from a breast cancer patient are summarised below.

**Figure 2.**
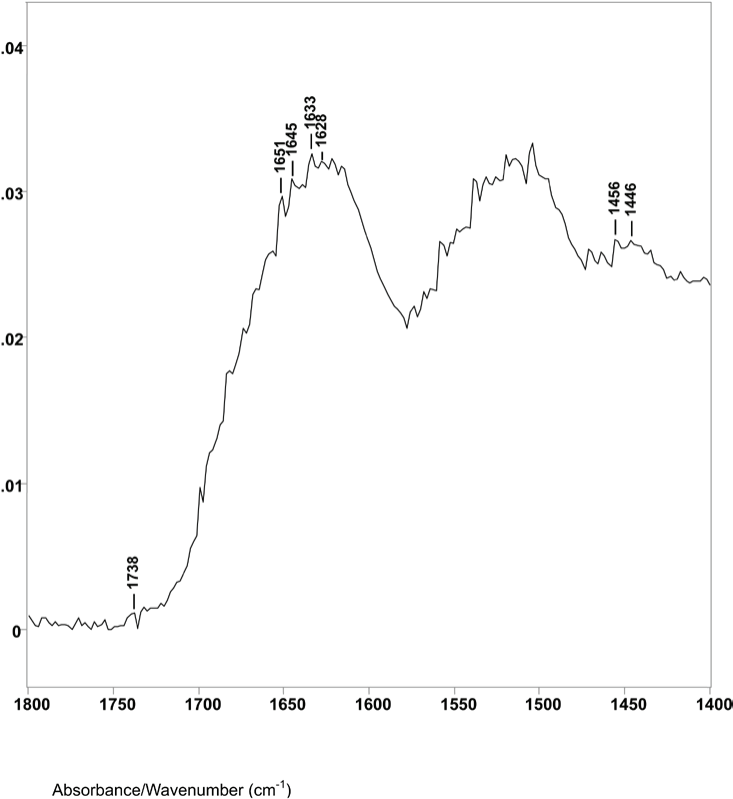
The 1800–1400 cm^−1^ region of difference spectrum A from 43° θ minus 45° θ ZnSe ATR–FT-IR spectra.

The difference spectrum A shown in Figure 2 (spectral slice 43°θ minus 45°θ), represents a 1.2 μm axial layer thickness of a spectral slice of the hair fibre longitudinally located between 4.1 and 5.3 μm from its surface. The spectrum shows the Amide I IR absorptions at 1651 cm^−1^ (helical), 1645 cm^−1^ (parallel beta sheet), 1635, 1633, and 1628 cm^−1^ (antiparallel beta sheet) with peak ratios of 1.00, 1.03, 1.07, 1.09 and 1.07. This is typical for alpha keratin IR absorptions of the elongated cortical cells in both cancer positive and negative hair fibres.

The difference spectrum B (spectral slice 45°θ minus 46°θ) representing a 0.37 μm axial layer thickness of the hair fibre spectral slice of the hair fibre longitudinally located between 3.7 and 4.1 μm from the surface. Its spectrum (not shown) represents a transition spectrum between spectral slice A and spectral slice C. While its IR absorptions are more similar to spectrum A, some absorptions show similarity to spectrum C. Spectral slice B would appear to be from the irregular interface region where the normal bulk, elongated heterocortical cells (which give the hair fibre its strength) abut the flattened, ortho-type cortical cells of the surface. The irregular nature of this boundary surface area between these two types of cortical cell layers seen in electron micrograph of the ultrathin section of a human hair [17], would be expected to give this type of IR spectrum showing the presence of both types of cortical cells. This type of irregular interface also allows the release of some of the mechanically induced strain between the cuticle layer and the central cortex core, while preventing gross slippage between them.

**Figure 3.**
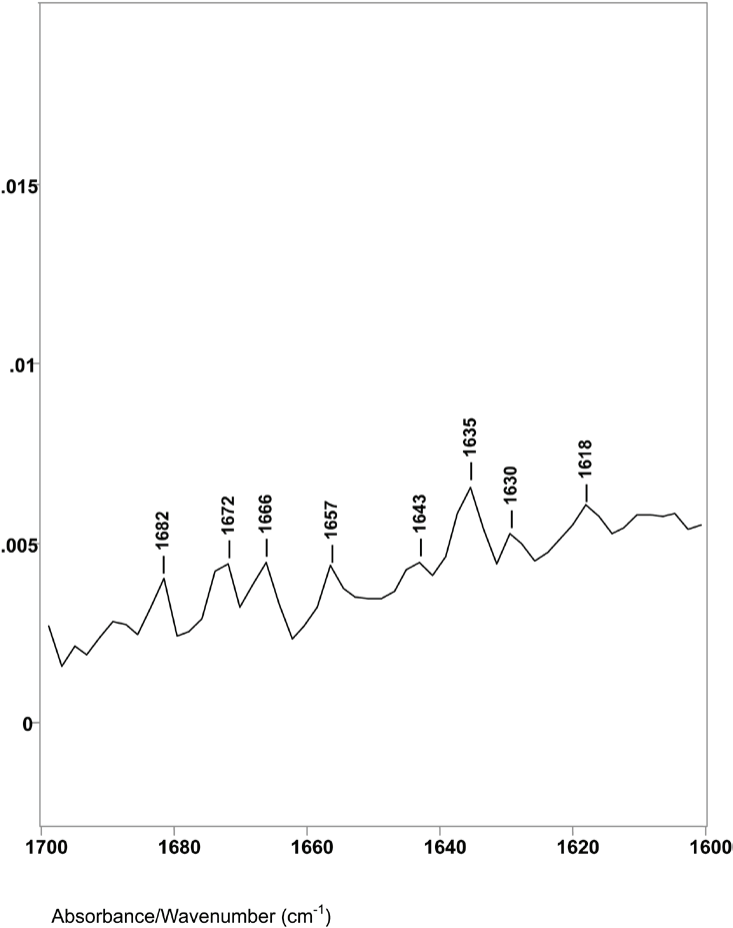
The 1760–1600 cm^−1^ region of difference spectrum C from 46° θ minus 47° θ ZnSe ATR–FT-IR spectra.

Difference spectrum C shown in Figure 3 (spectral slice 46°θ minus 47°θ), represents a 0.30 μm axial layer thickness of a spectral slice of the hair fibre longitudinally located between 3.4 and 3.7 μm from its surface. The spectrum shows major changes in the Amide I IR absorptions. There were no helical absorptions at 1651 cm^−1^ and there were changes in the types and amounts of parallel and antiparallel beta sheet absorptions. These peaks were at 1643, 1635, 1629 and 1618 cm^−1^ with peak height ratios of 1.0, 1.5, 1.3 and 1.4. The increases in peak height ratios and changes in peak positions are indicative of major changes in the molecular structure of keratins in this region of the hair fibre. While it is possible that the 1618 cm^−1^ absorption band could be related to an increase in side-chain C=C absorptions from tyrosine or tryptophane, it could be from denatured beta sheet structures.

Others have also reported that a narrow margin of the cortex adjacent to the cuticle stains well with osmonium tetroxide/lead acetate, whereas the bulk of the cortex did not [27] and that this narrow margin region appeared to be composed of concentric rings of tangentially angled intermediate filaments [28]. Thus, the molecular structure of proteins that make up the flattened, orthocortical cells that form the periphery of the cortex, that is, the region of DTZ, are different from the proteins in the heterotype cortical cells. It would appear they are formed from the cells that produce the keratins K31, K35, K85 and possibly K81 [29, 30].

The lipid infrared absorptions also changed in these spectral slices. Difference spectrum A showed ester absorption bands at 1738, 1734 and 1732 cm^−1^. The ratio of the C−H bending peak height at 1446 cm^−1^ to 1456 cm^−1^ was 0.85. This indicates that spectral slice A consists of bulk cortical cells and that the increase in breast cancer–related lipids is not located in this region. This is consistent with X-ray diffraction studies that indicate no change in the basic alpha keratin pattern of the macrofibrils of the cortex due to breast cancer [5–7, 24–26].

**Figure 4.**
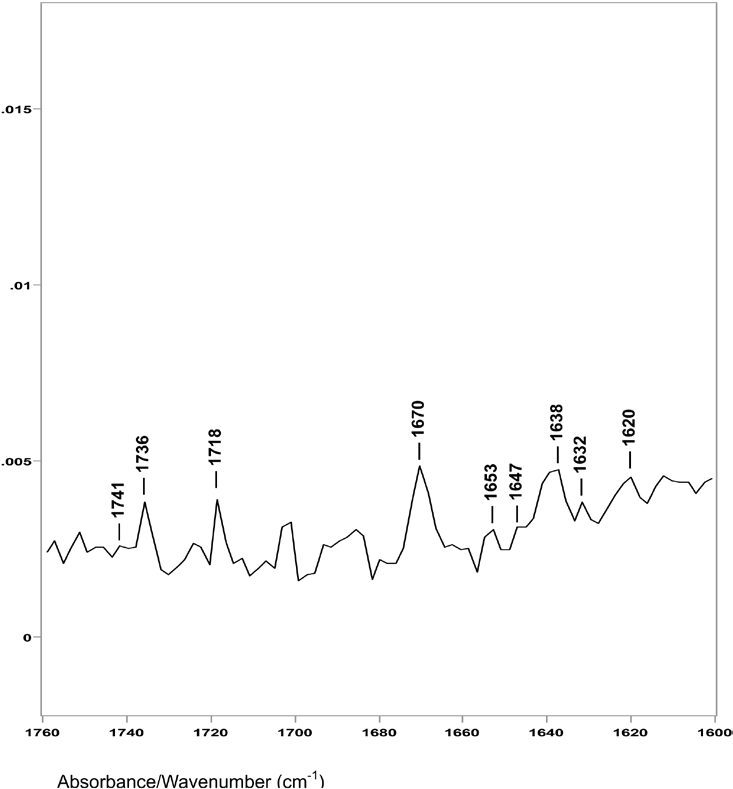
The 1760–1600 cm^−1^ region of difference spectrum D from 47° θ minus 48° θ ZnSe ATR-FT-IR spectra.

However, difference spectrum D shown in Figure 4 (spectral slice 47°θ minus 48°θ), represents a 0.22 μm axial layer thickness of material located about 3.2 μm from the hair fibre surface. This difference spectrum shows an increase in the ratio of the C−H peak height bending absorptions from less than 1.0 to about 1.45. The ester absorptions are at 1749, 1745, 1741 and 1736 cm^−1^ (with peak height ratios of 1.00 to 1.01 to 1.02 to 1.51). This increase in the C−H bending peak height absorption ratio, the disappearance of the 1738 and 1732 cm^−1^ ester peaks, and the appearance of a new ester peak at 1736 cm^−1^ indicate that this is the DTZ is the area of the hair fibre structure where increased lipid material associated with breast cancer is detected, both in our ATR–FT-IR spectroscopy and in the synchrotron X-ray diffraction studies. It is interesting to note that oleic and palmitic fatty acid esters of cholesterol have IR absorptions around 1736 cm^−1^. In addition, the C−H absorptions at 1466 and 1377 cm^−1^ in these hair samples are also related to cholesterol esters of oleic and palmitic fatty acids.

The spectral slice 48°θ minus 49°θ representing a 0.2 μm axial layer thickness of material immediately under spectral slice D could be overlaid with the absorption peaks of spectral slice C, indicating a similarity in structure. Thus, this material still represents a portion of the flattened orthocortical cells.

## Conclusion

There is a discrete transition zone (DTZ) between the hard protective cuticle layer and the softer elongated cortical cells of the cortex. The DTZ is composed of flattened orthocortical cells located on the outer perimeter of the cortex and appears to range in thickness between 2 and 3.6 μm. The inner surface of the DTZ, adjacent to the elongated cortical cells that make up the core of the hair fibre, is irregular.

ATR–FT-IR analyses of these flattened orthocortical cells indicate major changes in the molecular structure of keratins found in this transition zone. Other studies have identified cells that produce keratins that are distinct from alpha keratins found in the elongated cortical cells in the hair fibre core. These distinct keratins appear to be produced in the lower region of the hair follicle at the interface between the cuticle and cortex [29, 30].

ATR–FT-IR has also identified the DTZ as the region where increases in lipid esters are indicative of breast cancer. When the breast cancer is clinically removed, both the 1736 cm^−1^ ester band absorption and the increase in the 1446 to 1456 C−H bending absorption ratio are no longer observed. This suggests that biomarkers produced by the breast cancer interact with stem or other cells near the lower region of the follicle, controlling the amount and type of lipid esters in the DTZ. These esters may be oleic and palmitic esters of cholesterol.

## Conflicts of interest

The authors state no conflict of interest.

## Figures and Tables

**Table 1.** Effective depth of penetration of evanescent wave of IR beam (in μm) for ZnSe IRE and Hair: Wavenumber (ν in cm−1) versus incident light angle (θ).

ν/θ	43°	44°	45°	46°	47°	48°	49°	50°	51°	52°	53°	54°	55°
3000	2.88	2.49	2.23	2.03	1.88	1.76	1.66	1.58	1.5	1.44	1.39	1.34	1.29
2800	3.08	2.67	2.39	2.18	2.02	1.89	1.78	1.69	1.61	1.54	1.49	1.43	1.39
1740	4.96	4.3	3.84	3.51	3.25	3.04	2.86	2.72	2.59	2.49	2.39	2.31	2.23
1640	5.26	4.56	4.08	3.72	3.44	3.22	3.04	2.88	2.75	2.64	2.54	2.45	2.37
1500	5.76	4.98	4.46	4.07	3.77	3.52	3.32	3.15	3.01	2.88	2.77	2.68	2.59
1400	6.17	5.34	4.77	4.36	4.03	3.77	3.58	3.38	3.22	3.09	2.97	2.87	2.77
1300	6.64	5.75	5.14	4.69	4.35	4.07	3.83	3.64	3.47	3.33	3.19	3.09	2.99
1200	7.19	6.23	5.57	5.08	4.71	4.4	4.15	3.84	3.76	3.61	3.47	3.34	3.24
1000	8.63	7.48	6.68	6.11	5.65	5.28	4.98	4.73	4.51	4.33	4.16	4.01	3.88
900		8.31	7.43	6.78	6.28	5.87	5.54	5.26	5.02	4.81	4.62	4.46	4.31
800			8.36	7.63	7.06	6.61	6.23	5.91	5.64	5.41	5.21	5.02	4.85
700				8.72	8.07	7.55	7.12	6.76	6.45	6.18	5.94	5.73	5.55
